# Application of the Generalized Maxwell Model for Single-Kernel Relaxation Experiments—Part 1: Effect of Wheat Type and Moisture Content

**DOI:** 10.3390/ma19122592

**Published:** 2026-06-16

**Authors:** Grzegorz Łysiak, Jawad Kadhim Al Aridhee, Ryszard Kulig, Paweł Sobczak, Renata Różyło, Artur Przywara

**Affiliations:** 1 Department of Food Engineering and Machines, University of Life Sciences in Lublin, 20-950 Lublin, Poland; pawel.sobczak@up.edu.pl (P.S.); renata.rozylo@up.edu.pl (R.R.); 2 College of Agriculture, Al-Muthanna University, Samawah 66001, Iraq; jawadaridhee@gmail.com; 3 Department of Machinery Operation and Production Processes Management, University of Life Sciences in Lublin, 20-950 Lublin, Poland; artur.przywara@up.edu.pl

**Keywords:** wheat, moisture, compression, stress relaxation, Maxwell model

## Abstract

This study aimed to characterize the stress relaxation of wheat kernels of different hardness and varying moisture contents using generalized Maxwell models with three, five, and seven elements. Single-kernel stress relaxation tests were conducted under controlled axial compression to obtain force decay data, which were fitted using non-linear regression to estimate model constants. Positive correlations between kernel moisture content and Maxwell constants were observed, indicating greater magnitude of force decay and higher relaxation rates at higher moisture levels, while the residual force decreased. Significant differences in viscoelastic parameters were also found between hard and soft wheat varieties.

## 1. Introduction

Agricultural and food products exhibit complex rheological behaviors, spanning a broad spectrum of mechanical responses [1]. While simplified models—such as ideally elastic (Hookean solids), ideally viscous (Newtonian fluids), and ideally plastic bodies—are often invoked for approximation, real food systems rarely conform to these idealized extremes.

Among various approaches to describe viscoelastic behavior, the generalized Maxwell model has proven especially effective for characterizing stress relaxation in diverse food matrices. The classic Maxwell model consists of a Hookean spring (elastic element) in series with a Newtonian dashpot (viscous element). In this configuration, the spring constant reflects the material’s rigidity, and the relaxation time (τ) quantifies the timescale over which stress decays via molecular rearrangements [2,3,4]. Shorter relaxation times generally indicate reduced elasticity and lower rigidity. However, this single-element Maxwell model does not accommodate equilibrium (long-term) stress, limiting its applicability for materials that retain residual stress under constant strain.

To capture both instantaneous and equilibrium responses, the generalized Maxwell model (GMM) introduces multiple Maxwell elements (each a spring and dashpot in series) arranged in parallel with one additional spring [5]. Under constant strain, the total stress is the sum of the stresses from each parallel element. Each spring–dashpot pair can be associated with specific structural components or relaxation mechanisms at the cellular or molecular level [6,7]. Sozer and Dalgic [3] proposed that the number of Maxwell elements required to fit relaxation data correlates with the underlying cellular architecture. Accordingly, a wheat kernel can be conceptualized as comprising zones with distinct decay rates and apparent viscosities, each represented by a Maxwell element.

GMMs with varying element counts have successfully described stress relaxation in numerous biological materials, including intact wheat kernels [7,8,9,10], rice [11], maize [12,13], cowpeas [14], soybeans [15], lentils [16], bread [4,17], wheat/starch gels, dough, and gluten systems [8,18,19]. It is well established that increasing the number of elements typically improves fit quality [7,16,20]. For example, a five-element Maxwell model (one elastic spring in parallel with two Maxwell bodies) effectively captured relaxation in lipids (e.g., beeswax, candelilla wax, and carnauba wax), high-melting milk fat fractions, and alginate gels [21,22]. Similarly, a five-parameter Maxwell model described potato tissue relaxation under pulsed electric fields [23] and accurately reproduced viscoelastic behavior in pork ham [24]. In maize seeds, the five-element configuration outperformed three-element models [13], whereas a seven-element model best fit the kinetics of maize tortilla staling [25]. The increase in the number of Maxwell elements generally improves the model’s ability to approximate experimental data [20]. However, this comes at the cost of higher model complexity and more parameters to estimate. There are insufficient data on how the model parameters themselves change.

Water content is a critical variable governing rheological properties. In liquids, moisture primarily influences viscosity and consistency; in solids, it modulates mechanical response. Increasing water content may induce a plasticizing effect (softening, increased extensibility, reduced hardness) or—less commonly—an antiplasticizing (toughening) effect (increased toughness, reduced brittleness) [10,14]. Lewicki [26] noted that moisture toughening can enhance material strength under certain conditions. Moisture-dependent shifts in relaxation spectra have been documented for wheat kernels [27,28], with relaxation curves shifting downward as moisture rises. Peleg–Normand model parameters also vary markedly with moisture in soft wheat [27,29]. Bargale & Irudayaraj [10] reported decreases in elastic moduli and relaxation times with increasing moisture in various legumes and cereals. Ozturk & Takhar [20] found that oat flakes with higher moisture relaxed more rapidly due to softening, yielding lower spring constants and shorter τ values. However, moisture effects can be complex: Bhattacharya [30] documented both increases and decreases in Maxwell parameters for bean flour dough, and chickpea seeds [31] exhibited time-dependent moisture influences.

Experimental conditions also affect estimated model parameters. Factors such as deformation level, strain rate, and data-fitting methodology introduce variability in the fitted constants [29,30]. Although the present study’s broader experimental design encompasses these aspects, herein we focus specifically on grain type and moisture content. Other dependencies will be addressed in subsequent publications.

In summary, the variability in GMM parameters arises from both intrinsic material properties and extrinsic testing conditions. This work extends previous studies on wheat kernel relaxation [28,29] by analyzing how model coefficients vary with moisture content and selected experimental procedures. Given the paucity of comprehensive data linking rheological model parameters to both material characteristics and test protocols, this article represents part of a larger effort to characterize and apply the viscoelastic properties of wheat grains under diverse conditions.

## 2. Materials and Methods

### 2.1. Material Selection

This study evaluated four wheat cultivars, selected to represent both soft- (*Triticum aestivum*) and hard-endosperm (*Triticum durum*) types. Two soft-endosperm cultivars—Zawisza and Wydma—are Polish varieties obtained from the Plant Breeding Station HR Smolice (Smolice, Poland). One hard-endosperm cultivar, SMH87, also originated from HR Smolice. SMH87 is the first Polish durum wheat variety adapted for cultivation in northeastern and central Europe. It is a wheat variety intended exclusively for the production of high-quality pasta. The second durum cultivar was obtained from the pasta manufacturer Lubella (Lublin, Poland), though more information on its origin and variety is not available. For clarity and consistency throughout the manuscript, the wheat varieties are designated as follows: Zawisza—SOFT1, Wydma—SOFT2, SMH87—HARD1, and wheat from Lubella company—HARD2.

The choice of both soft and hard wheat types was intentional, based on their well-documented differences in mechanical properties, particularly in terms of stress–strain behavior and susceptibility to fragmentation during mechanical testing. The hard and soft wheat varieties used in this study differed significantly in key quality parameters such as protein content, starch content, wet gluten content, and the Zeleny index (Table 1). These parameters were determined in the CLA laboratory (the University of Life Sciences in Lublin, Poland) using its approved analytical methods.

### 2.2. Stress Relaxation Testing: Equipment and Procedures

Stress relaxation experiments were conducted in the Department of Equipment Operation and Maintenance in the Food Industry at the University of Life Sciences in Lublin, Poland. A Zwick Z020 universal testing machine (Zwick/Roell GmbH & Co. KG, Ulm, Germany), equipped with testXpert^®^ software (version 7.1), was used to perform the measurements under controlled conditions.

**Figure 1:**
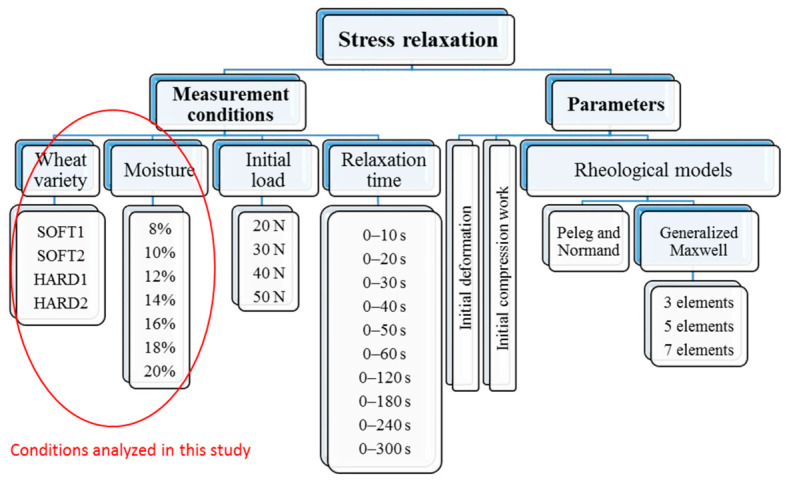
Scheme of the experimental plan.

The overall experimental framework is outlined in Figure 1. It is important to note that the influence of two parameters—initial compression force and relaxation time—will be addressed in detail in future publications. For the current study, the focus was limited to evaluating the effects of wheat type and moisture content. Nonetheless, all recorded measurements, across various force levels and relaxation durations, were included in the statistical analysis to enhance the robustness and generalizability of the observed trends.

The impact of the initial load and relaxation time will be analyzed in future work. Consequently, the data presented here on the influence of wheat type and moisture content include all individual measurements across all load levels and relaxation times, enhancing the robustness and statistical power of the observed relationships.

#### 2.2.1. Moisture Determination

For each wheat cultivar, the initial moisture content was determined in accordance with the PN-EN ISO 712:2012 standard. Three replicates of 5 g samples were dried at 130 °C for 2 h. The moisture content (MC, wet basis) was calculated using the following formula:

(1) $$\mathrm{MC}\;=\;\frac{M_{w}-M_{d}}{M_{w}}\cdot 100\%$$

where (M_w_) and (M_d_) represent the weights of the sample before and after drying, respectively.

The experimental design included seven target moisture levels: 8%, 10%, 12%, 14%, 16%, 18%, and 20% (wet basis, w.b.). To achieve these levels, a wheat batch with a known initial moisture content and weight was dried at 40 °C until it reached the lowest target moisture content (8%). Subsequently, the batch was subdivided into seven sub-samples, and calculated amounts of distilled water were added to each to attain the desired moisture contents based on mass balance calculations.

The conditioned samples were stored in airtight containers at 8 °C for 48 to 72 h and periodically mixed to ensure uniform moisture distribution. The final moisture content was verified after 48 h; only samples within ±0.2% of the target moisture levels were selected for subsequent testing. Prior to the relaxation tests, the samples were removed from refrigeration and equilibrated at ambient temperature for approximately 30 min.

#### 2.2.2. Relaxation Tests

Individual wheat kernels were placed on the testing platform of the Zwick Z020 machine with their ventral side down, ensuring consistent orientation across all trials. A capture with maximum capacity of 100 N and accuracy class of 0.5 was used. This means the error of force measurement was 0.5%. Each kernel was axially compressed at a constant loading rate of 10 mm/min until a predetermined initial force level was reached. Four initial load levels were used: 20, 30, 40, and 50 N. The upper limit (50 N) corresponded to approximately 50% of the average rupture force of a wheat kernel, thereby ensuring structural integrity during relaxation measurements. Upon reaching the target load, the deformation (strain) was held constant for a duration of 300 s. During this relaxation phase, the decay of the compressive force over time was recorded using the testXpert^®^ software at a sampling frequency of 10 Hz.

For each test condition (moisture level × load level × variety), 20 grains were tested to ensure the statistical meaning of the results.

### 2.3. Modeling of Stress Relaxation Using Generalized Maxwell Models

The experimentally obtained stress relaxation curves were analyzed using the generalized Maxwell model (GMM). This model captures the characteristic stress decay over time following a constant strain deformation and is particularly suited for materials that exhibit both elastic and viscous behavior.

**Figure 2:**
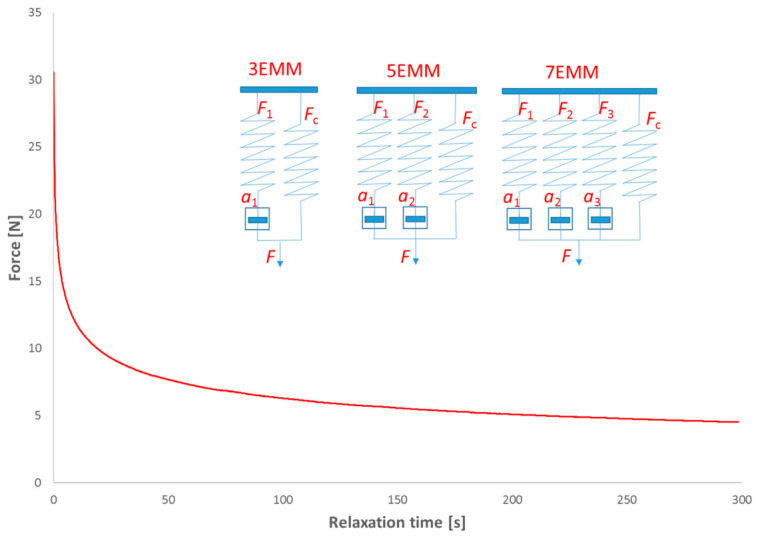
Example of a relaxation curve and the configuration of the applied Maxwell models.

To assess the effect of model complexity on fitting accuracy, three variants of the generalized Maxwell model were implemented (Figure 2):

(1) Three-element Maxwell model (3EMM): One Maxwell element (a spring and dashpot in series) in parallel with a free (equilibrium) spring;
(2) Five-element Maxwell model (5EMM): Two Maxwell elements in parallel with a free spring;
(3) Seven-element Maxwell model (7EMM): Three Maxwell elements in parallel with a free spring.

It is worth noting that in the literature, two naming conventions are often used interchangeably: (i) according to the number of Maxwell elements (e.g., one-, two-, and three-element Maxwell systems), and (ii) according to the total number of mechanical elements (e.g., three-, five-, and seven-element models including the equilibrium spring). Since most food materials exhibit a residual (non-zero) stress after long-term deformation [20], all tested models included an equilibrium (free) spring to capture this behavior.

The mathematical expressions for the tested models are presented below:

Three-element Maxwell model:

(2) $$F\left(t\right)=F_{1}{exp}^{-a_{1}t}{+F}_{c}$$

Five-element Maxwell model:

(3) $$F\left(t\right)=F_{1}{exp}^{-a_{1}t}+F_{2}{exp}^{-a_{2}t}+F_{c}$$

Seven-element Maxwell model:

(4) $$F\left(t\right)=F_{1}{exp}^{-a_{1}t}+F_{2}{exp}^{-a_{2}t}+F_{3}{exp}^{-a_{3}t}+F_{c}$$

where F(t) in N represents the magnitude of the force decay; F_c_ is a single spring constant (equilibrium force); F_1_, F_2_, and F_3_ and *a*_1_, *a*_2_, and *a*_3_ are the constants representing three term Maxwell elements; and where *a*_i_ = 1/τ_i_, i = 1, 2 or 3.

The relaxation time of the Maxwell element τ_i_ is also expressed as the time required for stress in an element to decay to 1/e (36.8%) of its original value.

**Figure 3:**
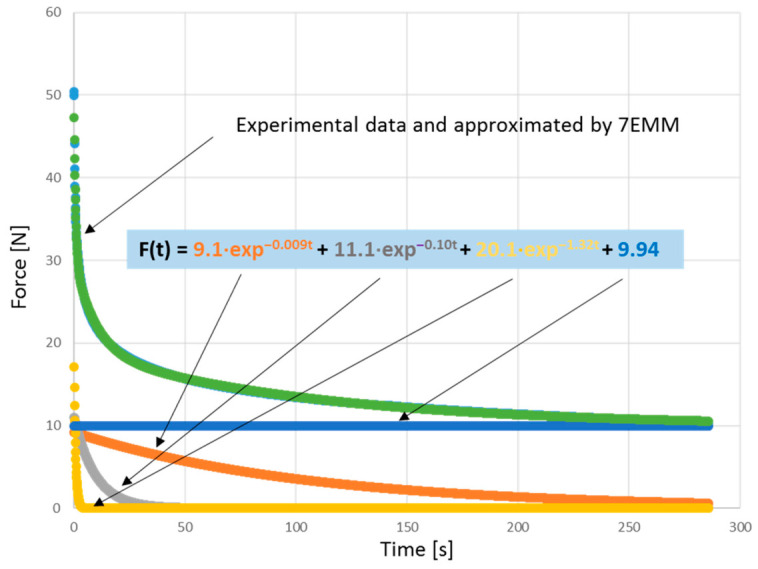
The interpretation of constants for the 7EMM Maxwell model, colors represent different Maxwell elements.

The interpretation of the model constants is presented in Figure 3, illustrating the combined effects of the individual pair spring–dashpot model elements. In this representation, the values of F_1_, F_2_, F_3_, and F_c_ reflect the contributions of the respective relaxation forces in the model, while the constants *a*_1_, *a*_2_, and *a*_3_ correspond to the relaxation times of these elements.

While characterizing biological materials using fundamental engineering properties like stress, strain, or Young’s modulus is theoretically optimal, its practical application to intact grain remains highly challenging. Due to the irregular geometry, structural heterogeneity, and anisotropy of wheat kernels, true stress calculations under varying moisture and deformation levels introduce a profound experimental dilemma. Testing isolated kernel fragments is an alternative; it introduces other practical uncertainties regarding sample preparation and representativeness. Consequently, testing intact kernels via force relaxation offers a reliable, highly reproducible alternative for quality classification and technological assessment, as demonstrated by standardized methods like the Single-Kernel Characterization System (SKCS) [32]. This kind of assumption was also used in many studies [33,34]. In this study, prioritizing force relaxation over stress simplified experimental procedures and streamlined the interpretation of the kernel’s response to axial loading, thereby providing potentially relevant data to optimize wheat classification or processing.

### 2.4. Calculations and Statistics

The estimation of model parameters for the viscoelastic behavior of wheat kernels was performed using non-linear regression based on the Levenberg–Marquardt algorithm, implemented in the Statistica software (version 13.1, TIBCO Software Inc., Palo Alto, CA, USA). This algorithm enables efficient curve fitting of multi-parameter, non-linear models and is widely employed in rheological modeling of biological materials. Its application in similar contexts has been previously documented in [4,6,34].

For selected conditions, the Root Mean Square Error (RMSE) of the analyzed models was determined based on the equation:

(5) RMSE=1n∑i=1nyi−y^i2

where *n*—the total number of observations, $y_{i}$—the observed value, and ${\hat{y}}_{i}$—the predicted value.

All statistical analyses were also conducted using Statistica 13.1. A significance level of 0.05 was applied in all evaluations. Descriptive statistics, analysis of variance (ANOVA), Tukey’s HSD, and regression diagnostics were used to assess the goodness of fit and to test the significance of the estimated model parameters across different wheat types and moisture levels. Statistical results (basic statistics and variance analysis) are provided in the Supplementary Material.

## 3. Results

Within the scope of this study (Figure 1), the variability in the models relative to wheat kernel moisture content and hardness was investigated, and their dependence on load magnitude and relaxation time was confirmed. The subsequent analyses considered all deformation levels and relaxation times simultaneously. This was the main reason for the relatively large standard deviations and coefficients of variation for the conditions and parameters tested. Standard errors, on the other hand, were within low limits (Tables S1–S3). While this approach influenced the model parameters as a function of kernel moisture content and hardness, the results provide an average representation across the examined loading conditions and relaxation times. Furthermore, this approach increased statistical power by substantially expanding the number of tests performed, models generated, and comparisons made.

### 3.1. Generalized Maxwell Three-Element Model (3EMM)

**Figure 4:**
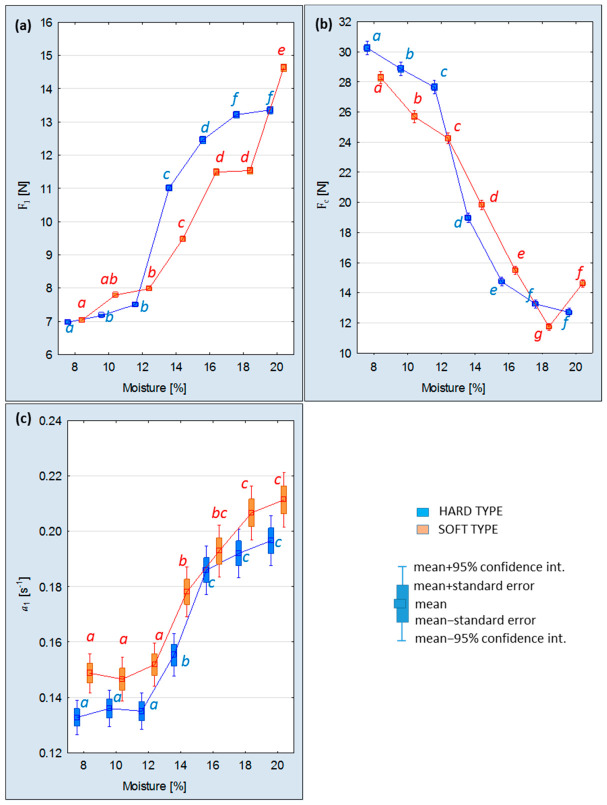
Influence of kernel moisture on the constants F_1_ (**a**), F_c_ (**b**), and *a*_1_ (**c**) of the Maxwell three-element model (letters represent homogeneous groups).

**Figure 5:**
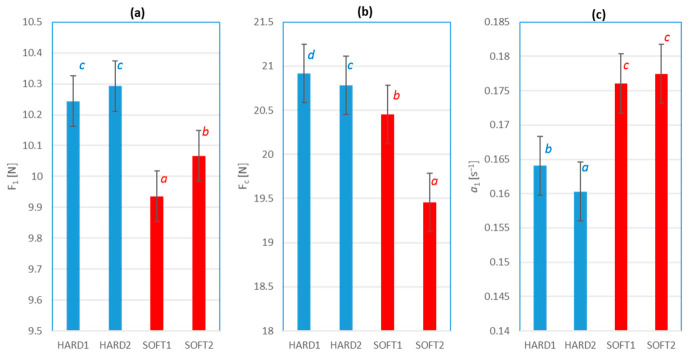
Influence of wheat variety on the constants F_1_ (**a**), F_c_ (**b**), and *a*_1_ (**c**) of the Maxwell three-element model (letters represent homogeneous groups).

The results of stress relaxation modeling using the three-element Maxwell model are presented in Figure 4 and Figure 5. In this model, parameter F_1_ represents the magnitude of the force component attributed to the first spring reflecting the initial portion of the force that undergoes relaxation, while *a*_1_ corresponds to its relaxation rate. Higher values of *F*_1_ indicate a greater initial force involved in relaxation, whereas higher values of *a*_1_ denote faster force decay (i.e., shorter relaxation time). The third parameter, F_c_, represents the residual (equilibrium) force at the end of the relaxation period.

A positive correlation was observed between moisture content and both *F*_1_ and *a*_1_. As the moisture content increased from 8% to 20%, the average value of *F*_1_ approximately doubled (from ~7.0 N to ~14.0 N). While an increase in F_1_ results in a greater decrease in force due to the initial relaxation phase, it must be accompanied by a corresponding decrease in the residual force F_c_. Accordingly, the residual force F_c_ exhibited a significant decrease with increasing moisture content, dropping from an average of 29.3 N (at 8% moisture) to 13.6 N (at 20% moisture). This indicates a lower proportion of force retained in the structure after the relaxation period, confirming the plasticizing effect of water on kernel texture. The results suggest that a greater proportion of the applied force was relaxed in more hydrated (i.e., softer) kernels. This trend reflects the increased deformability and reduced internal resistance of kernels at higher moisture levels.

Moreover, it was observed—and statistically confirmed—that at low moisture levels, the value of the constant F_1_ was slightly higher for soft-textured cultivars, whereas at higher moisture contents (with the exception of 20%), it was higher for hard-textured ones. Similarly, the F_c_ force values at low moisture levels were lower for soft cultivars and, conversely, higher for hard cultivars. From a methodological standpoint, an important conclusion emerging from the above analysis highlights the influence of moisture on the interrelationship between the model constants. Correspondingly, *a*_1_ increased from approximately 0.14 to 0.20 s^−1^, with only slight variation for the 8–12% moisture range, and a more pronounced increase observed at higher moisture contents. This indicates a faster stress relaxation rate in more hydrated kernels, consistent with their plasticized mechanical behavior. Across the entire range of tested moisture contents, the parameter *a*_1_ was consistently higher for soft cultivars.

The highest values of F_1_ and F_c_ were recorded for hard wheat cultivars (HARD1 and HARD2), while the lowest values were observed for soft ones (SOFT1 and SOFT2). The differences in F_1_ between varieties were statistically significant (*p* < 0.05), indicating varietal influence on the elastic force components. Despite the confirmation of significant differences, it should be noted that moisture content—as indicated above—is a critical parameter that may alter this pattern. In this context, a moisture level of nearly 12–14% appears to constitute a threshold for change. The parameter *a*_1_ showed lower variation between varieties and no significant differences were found between the soft and hard wheat types. The relaxation time, characterized by the constant *a*_1_, exhibited significant variation depending on the grain endosperm structure and was notably shorter for soft-textured grain cultivars.

The coefficients of variation (CVs) for each of the three model constants are reported in Supplementary Table S1. For both spring-associated constants (F_1_ and F_c_), the CV increased with moisture content, while *a*_1_ demonstrated the highest variability among the three parameters. In terms of varietal differences, hard-type wheats exhibited, on average, ~5% higher variability in model parameters compared to soft types. Interestingly, the variability in *a*_1_ only slightly changed with moisture content.

Tukey HSD comparisons revealed that grain hardness had a highly significant effect on all 3EMM constants (*p* < 0.0001).

### 3.2. Generalized Maxwell Five-Element Model (5EMM)

**Figure 6:**
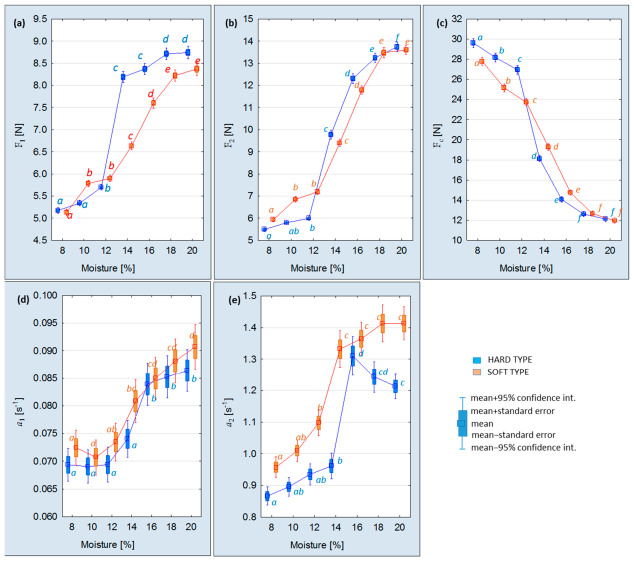
Influence of kernel moisture content on the constants F_1_ (**a**), F_2_ (**b**), F_c_ (**c**), *a*_1_ (**d**), and *a*_2_ (**e**) of the Maxwell five-element model (letters represent homogeneous groups).

To achieve a more accurate representation of the viscoelastic behavior of wheat kernels during stress relaxation, a five-element Maxwell model was applied. Figure 6 shows the effect of moisture content of wheat on the constants of the five-element Maxwell model: F_1_, F_2_, F_c_, *a*_1,_ and *a*_2_. F_1_ and F_2_ refer to the magnitude of force decay expressed by first and second spring, whereas constants *a*_1_ and *a*_2_ represent the rate of force relaxation.

Similarly to the case of the 3EMM, higher values of F_2_ correspond to larger amounts of initial force relaxed, and higher values of *a*_2_ are attributed to the shorter relaxation periods. The same is represented by constants F_1_ and *a*_1_, but the time scale is longer. The second spring reveals the first and short relaxation period (because *a*_2_ >> *a*_1_), whereas the first spring reveals the continuous stage of the process. F_c_ represents the equilibrium of the residual force at the end of the experiment.

Positive correlations between moisture content of wheat and constants F_1_ and F_2_ were obtained. With an increase in the moisture, the value of constants increased successively. Average values of F_1_ and F_2_ increased from 5.1 and 5.7 to 8.5 and 13.6, respectively. Significant differences between the means for each moisture level were confirmed. This indicates a growing capacity of kernels to undergo relaxation under compression, particularly in their plastic state. The distribution of the results of F_1_ and F_2_ were found to be narrower for drier samples. For the third constant, F_c_, a significant decline from 28.69 to 12.04 was observed with the increasing moisture content. The averages were also statistically different for all moisture levels. This suggests that hydrated kernels retain less elastic force after relaxation, consistent with a softer, more compliant structure. The coefficient *a*_1_ increased from about 0.070 to 0.088. For relatively dry samples (from 8 to 12%), it changed only slightly. Constant *a*_2_ increased from 0.91 to 1.34 with increasing moisture content. The means were not statistically different for 16, 18, 12 and 20%. The increase in *a*_1_ and *a*_2_ indicates a faster decline of loading force for softer kernels. A positive correlation between the two constants was observed, hence a higher speed of force relaxation at the initial phase was followed by a similar effect during the next stage.

For both short and long relaxation periods, the relaxed force values in the moisture range of 8–12% were higher for soft wheat varieties. Conversely, in the moisture range of 14–20%, the opposite situation was observed, particularly for F_1_ (long relaxation period). In the case of F_2_, the values were very similar. This also highlights a distinct shift in the relative values between the wheat types within the moisture range of approximately 12–14%, which was also characteristic of the 3EMM. Moreover, this shift was more pronounced for hard wheat, as evidenced by a marked increase in the F_1_ and F_2_ parameters and a decrease in F_c_. When comparing the values of F_1_ and F_2_, it was observed that a greater proportion of the relaxed force for both wheat types occurred during the initial short phase of the process (F_2_ > F_1_). The constants *a*_1_ and *a*_2_ increased across the entire tested moisture range, indicating shorter relaxation times at higher moisture levels. Both constants were higher for the soft wheat varieties.

**Figure 7:**
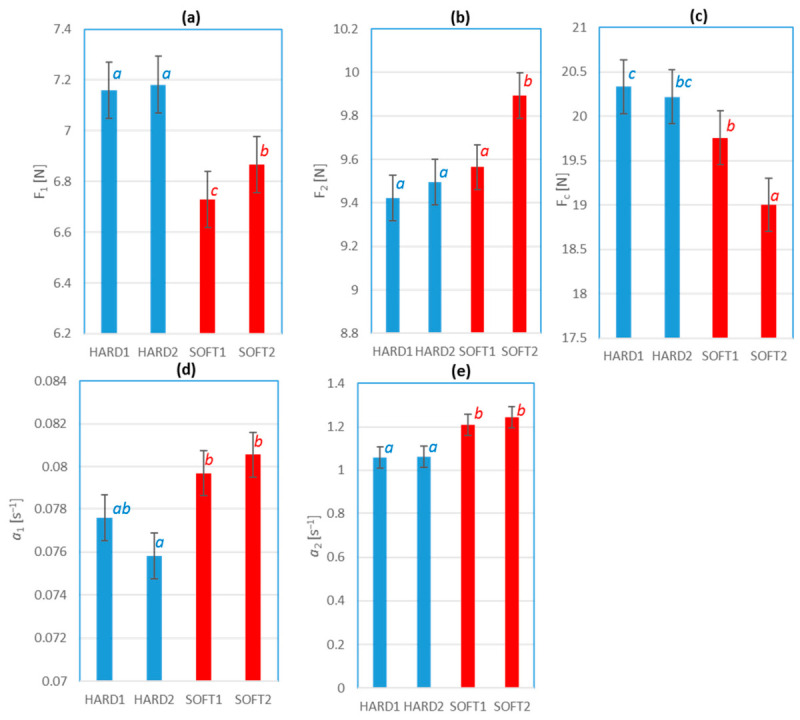
Influence of wheat variety on the constants F_1_ (**a**), F_2_ (**b**), F_c_ (**c**), *a*_1_ (**d**), and *a*_2_ (**e**) of the Maxwell five-element model (letters represent homogeneous groups).

Figure 7 shows the dependency of constants F_1_, F_2_, F_c_, *a*_1_, and *a*_2_ on wheat variety. For the first Maxwell element (long time effect), higher force relaxation magnitude was observed for hard wheat cultivars. On the other side, the rate of relaxation expressed by *a*_1_ was found to be smaller for them. However, the second element (short time effect) was characterized by slightly smaller values of the constant F_2_ for hard wheat varieties. The constants *a*_1_ and *a*_2_ were higher for soft-type endosperm, indicating shorter relaxation time. Finally, the residual and not-relaxed force was found to be higher for hard wheats. Though a slight difference between the means of all the model constants was observed, the means were statistically different between soft and hard kernels.

Data on the coefficients of variability of the five-element model were included in Table S2 (Supplementary). For this model, the coefficient of variation as a function of moisture content increased for the constant F_c_. In contrast, the variability in the constants F_1_ and F_2_ showed no clear relationship with moisture content. Similarly, it is difficult to infer the variability in the constants *a*_1_ and *a*_2_ both as a function of moisture content and wheat type. Compared to the 3EMM, the constants *a*_1_ and *a*_2_ showed lower variability.

Tukey HSD comparisons revealed, for the 5EMM, that all constants were significantly affected by wheat type (*p* < 0.0001), with the highest *p*-value observed for *a*_1_ (*p* = 0.0005).

### 3.3. Generalized Maxwell Seven-Element Model (7EMM)

**Figure 8:**
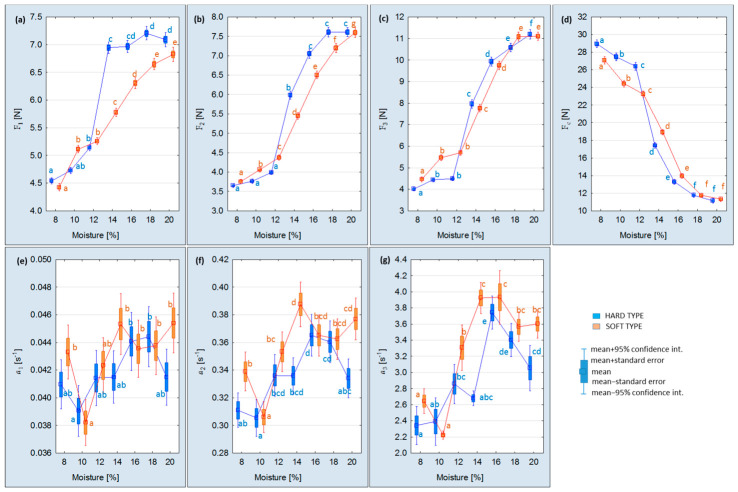
Influence of kernel moisture content on F_1_ (**a**), F_2_ (**b**), F_3_ (**c**), F_c_ (**d**), *a*_1_ (**e**), *a*_2_ (**f**), and *a*_2_ (**g**) of the Maxwell seven-element model (letters represent homogeneous groups).

Figure 8 shows the effect of moisture content on constants F_1_, F_2_, F_3_, F_c_, *a*_1_, *a*_2_, and *a*_3_. F_1_, F_2_, and F_3_ refer to the magnitude of force decay expressed by the first, second and third spring, whereas constants *a*_1_, *a*_2_ and *a*_3_ represent the rate (time) of relaxation by the corresponding dashpots. The third spring reveals the shortest relaxation period, while the first and the second show the continuous phases of the process. F_c_ represents the equilibrium of the residual force at the end of experiment.

Positive correlations between kernels’ moisture content and constants F_1_, F_2_ and F_3_ were observed. Average values of F_1_, F_2_ and F_3_ increased from 4.4, 3.7 and 4.2 to 6.9, 7.6 and 11.1, respectively. Significant differences between the means for each moisture level were confirmed. The distribution of experimental results was narrower for drier samples. For the residual spring constant, F_c_, a significant decline from 28.02 to 11.28 caused by the increasing moisture was perceived. The means were also statistically different at all studied moisture levels.

Similarly to the previously analyzed models, for all relaxation periods, the relaxed force values in the moisture range of 18–12% were higher for soft wheat varieties. Conversely, in the moisture range of 14–20%, the opposite trend was observed, particularly for F_1_ (long relaxation period). In the case of F_2_—and even more so for F_3_—the values were very similar. A distinct shift in the relative values between the wheat types was also noted within the moisture range of approximately 12–14%. However, in this case, for soft wheat varieties, a clear increase in relaxation force was observed after exceeding 12% moisture, which was more pronounced for shorter relaxation times.

When comparing the values of F_1_, F_2_, and F_3_, it was observed that the shorter the relaxation time, the greater the proportion of relaxed force (F_3_ > F_2_ > F_1_). This trend was characteristic of both wheat types.

The variability in the constants *a*_1_, *a*_2_, and *a*_3_ in the analyzed model was not as evident as in the 3EMM and 5EMM. The constant *a*_1_ increased only slightly, from approximately 0.039 to 0.044. The constant *a*_2_ increased from 0.31 to 0.36 with increasing moisture content. The constant *a*_3_ also increased from 2.30 to 3.84. The homogeneous groups are presented in the figure. All constants were higher for soft wheat varieties.

**Figure 9:**
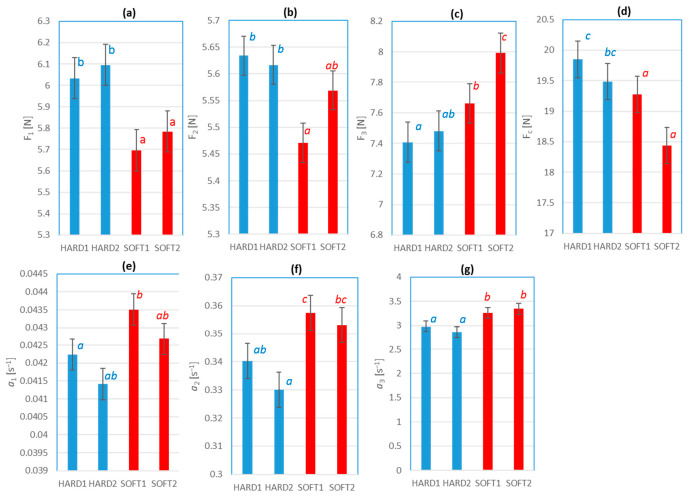
Influence of wheat variety on the constants F_1_ (**a**), F_2_ (**b**), F_3_ (**c**), F_c_ (**d**), *a*_1_ (**e**), *a*_2_ (**f**), and *a*_2_ (**g**) of the Maxwell seven-element model (letters represent homogeneous groups).

The spring and dashpot constants of the seven-element model differed depending on wheat variety (Figure 9). Similarly to the five-element model, for relatively longer relaxation times, scales of the magnitude of force decays (F_1_ and F_2_) were higher for hard wheats. On the contrary, for the shortest relaxation time represented by the third spring (F_3_), the force decay was found to be statistically lower for hard wheats than for soft wheats. The residual and not relaxed force, F_c_, was slightly higher for hard-variety endosperm. The rates of relaxation for all the Maxwell models were higher (lower *a*_1_, *a*_2_, and *a*_3_) for soft-endosperm types. The observed homogeneous groups are shown in the figure. It was perceived that with an increasing number of terms in the Maxwell model, the differences between constants for relatively longer time effects became smaller. This was true for both spring and dashpot constants.

By comparing the coefficients of variation in the model parameters (Table S3), it can be seen that there are no visible and clear dependencies on moisture content and wheat type. The variability was at similar levels as that of less complex models. In addition, no correlations were found between the variability in individual model parameters. However, it must be remembered here that the analysis of variability also includes the effect of initial loading and relaxation time.

Similarly to the previously analyzed models, for the 7EMM, significant differences across wheat type were confirmed for all parameters; the significance level was *p* = 0.02 for the *a*_1_ constant, *p* = 0.001 for F_2_, and *p* < 0.0001 for all remaining variables.

Interestingly, increasing the complexity of the Maxwell model (from three to seven elements) reduced differences between constants for longer relaxation times, both for spring and dashpot parameters. This suggests that more complex models capture viscoelastic behavior with finer detail, smoothing out apparent differences.

### 3.4. A Brief Comparison of Model Accuracy

**Figure 10:**
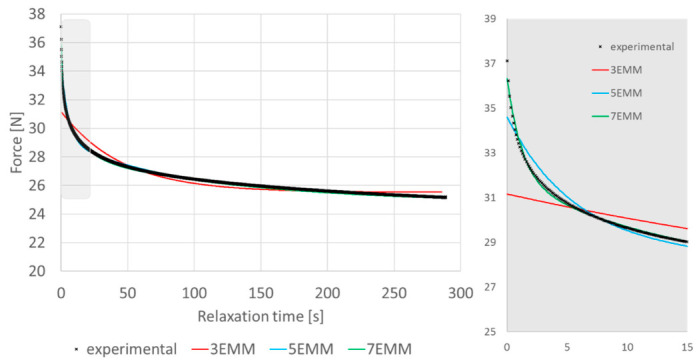
Relaxation curves obtained from experiments and estimated using models—3EMM (three-element Maxwell model), 5EMM (five-element Maxwell model), and 7EMM (seven-element Maxwell model) (wheat: HARD2, MC = 8%, load = 30 N, relaxation time = 300 s; the enlarged section highlights the initial relaxation phase).

Examples of model fittings are presented in Figure 10. The good and very good fit of the models to the experimental data somewhat obscures the picture presented in Figure 10. For the 3EMM, however, there are clear discrepancies between the model characteristics and the experimental data. Depending on the relaxation time, the predicted values were both higher and lower compared to the observed data. The 7EMM showed the best fit. The greatest differences between the models and quality of the fit and the experimental data occurred in the first few seconds of the relaxation (see selected details).

The relationship between predicted and observed relaxation force values as well as examples of residual distributions for the three studied models are shown in the following figure.

**Figure 11:**
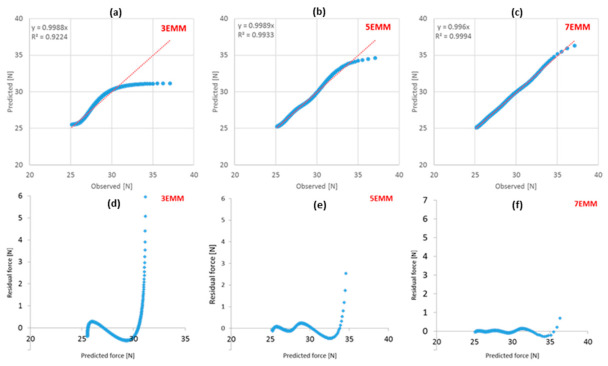
Predicted vs. observed relaxation force values (**a**–**c**) and residual analysis (**d**–**f**) for the applied Maxwell models (three-element Maxwell model, 5EMM, and 7EMM; wheat: HARD2, MC = 8%, load = 30 N, relaxation time = 300 s).

Figure 11a indicates that for the three-element Maxwell model, the predicted maximum relaxation forces during the initial phase are significantly lower than the observed values. As the relaxation time progresses, this trend reverses, only to shift again in the final relaxation phase. In contrast, for the 5EMM (Figure 11b), deviations are considerably smaller and primarily noticeable only during the initial relaxation phase. For the 7EMM (Figure 11c), these differences are practically negligible. This observation is further supported by the residual analysis (Figure 11d–f), which reveals asymmetry and non-random scatter for the three-element model, indicating an inadequate fit. In comparison, the 5EMM and 7EMM exhibit markedly improved accuracy, with residuals demonstrating increased symmetry and randomness. Although the 7EMM provides an excellent fit, a slight dependence of residuals on force magnitude, and consequently on relaxation time, remains slightly detectable. These overall trends in fit quality among the models were consistent across other testing conditions.

Table 2 presents the average RMSE values and coefficients of determination (R^2^) depending on wheat grain moisture content (load 30 N, relaxation time 300 s). The results shown in the table refer to models averaged for the HARD2 wheat variety under the above test conditions, based on 20 repetitions each.

It was observed that the RMSE decreased with increasing moisture content. This may relate to the fact that mechanical test results for dry or brittle materials are less repeatable compared to moist materials. On the other hand, the differences in error between models are relatively small, primarily because the main differences occur in the first seconds of relaxation. Therefore, it can be expected that shortening the relaxation time would increase the variability in the error in the estimated models.

Additionally, for the above conditions, an average relaxation curve was generated from 20 individual repetitions, and the evaluated models were applied to it. In this case, both RMSE and R^2^ values were significantly better than those presented in Table 2. RMSE ranged from approximately 0.041 to 0.087, representing less than 1% of the mean value. Higher determination coefficients were also obtained. These results simultaneously confirmed the observations presented above about the accuracy of the fits for the analyzed models.

Relating the accuracy of the analyzed models to grain moisture content, it can be concluded that, at higher seed moisture levels, where shorter force relaxation times have been observed, models such as the 7EMM are expected to provide higher predictive accuracy due to their superior performance in the short relaxation time range. Likewise, in the case of soft wheat cultivars, shorter relaxation times are more accurately captured by models comprising a greater number of elements.

## 4. Discussion

Analyzing the relaxation curves, it is evident that increasing moisture content leads to a greater and faster decrease in loading force. Shorter relaxation times characterize less elastic materials, which Sozer and Dalgic also note are easier to deform [3].

This study confirmed positive correlations between kernel moisture content and the Maxwell model constants across three-, five-, and seven-element configurations. As moisture content increased, so did the spring constants (F_1_, F_2_, F_3_) and relaxation rates (*a*_1_, *a*_2_, *a*_3_), indicating a more pronounced and faster decay in the loading force. These findings are consistent with previous research showing that increased moisture softens kernels, resulting in faster stress relaxation and greater deformability [2,3,9,20,35]. Some studies report a decrease in the spring constants at higher moisture levels [20,36]. In contrast, our results show increased spring constants for wetter kernels. This difference may result from the fact that our experiments applied a constant load magnitude across different moisture levels. Consequently, moist kernels experienced significantly greater deformations compared to dry ones. It is important to note that the components of the model change with varying load levels, and the sum of the coefficients corresponds to the total load magnitude over time. This highlights the importance of experimental conditions when interpreting rheological data and comparing results across studies.

Conversely, the residual force (F_c_) significantly decreased with higher moisture levels. A decrease in the F_c_ force was also observed by Ozturk for oat flakes [20]. A similar observation was made by Liu et al. [35] for soybean cotyledons.

In comparison, values of the constants *a*_1_, *a*_2_ and *a*_3_ increased as moisture content increased. Higher-moisture-content samples relaxed faster than lower ones. This trend was more pronounced for models with a smaller number of elements. It was observed in [20].

Relaxation times were shorter for soft-endosperm types. This finding is consistent with [37], where the authors reported shorter relaxation times for soft wheat compared to bread and durum wheats. Other authors have also indicated diverse relationships between model constants and kernel quality attributes. In our study, hard endosperm cultivars exhibited higher initial and residual forces and slower relaxation rates compared to soft types, indicating greater resistance to deformation and longer relaxation times.

For all models, it was found that the values of constants *a*_1–3_ were higher for soft wheat varieties. Considering that 1/*a* corresponds to the relaxation time (τ), it was concluded that the relaxation times were longer for hard varieties. This was also observed in [38]; however in our case, the differences were not as significant. For the 7EMM, the relaxation times were 24 s for the first element, 3 s for the second, and 0.3 s for the third. For the 5EMM, the relaxation times were approximately 13 s for the first period and 0.9 s for the shorter one. In the case of the 3EMM, the relaxation time was approximately 6 s.

It is important to emphasize two key observations from the conducted research and analysis—findings that have not been reported in any previous studies.

(1) When comparing the values of the constants F_1_, F_2_, and F_3_ for hard and soft wheat varieties in the low moisture range, the constants were higher for soft wheat; however, at higher moisture levels, they were lower compared to those of durum wheat.
(2) In the moisture range of 12–14%, a distinct increase in the constants was observed, particularly pronounced for durum wheat varieties. From a methodological standpoint, the variability in mechanical response within this moisture range may offer valuable insights for varietal comparisons. However, due to this variability, drawing conclusions based on single-point measurements may be unreliable.

Based on the trends of the analyzed parameters, the most effective differentiation between wheat varieties was achieved at moisture levels up to 12%.

Interesting results were provided by the analysis of relationships between the model parameters. The results for the 5EMM and 7EMM are presented in Table 3 and Table 4, respectively. The 3EMM was excluded from this analysis.

**Figure 12:**
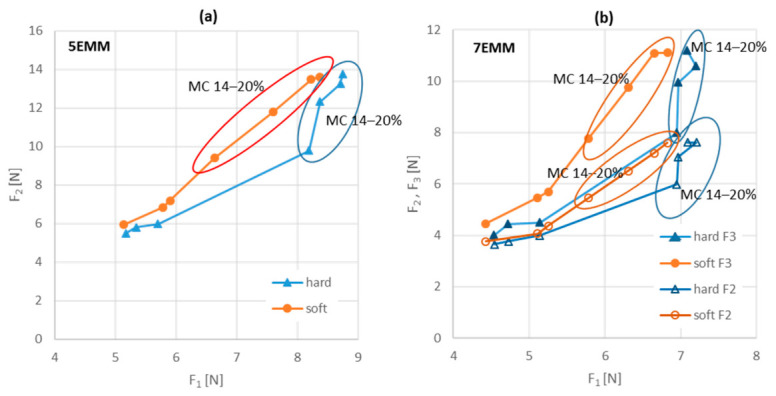
The relationship between the values of relaxed force for long and short relaxation times (**a**) 5EMM, (**b**) 7EMM.

For both the 5EMM and 7EMM, correlation analysis confirmed significant linear relationships between all model parameters, with the exception of F_1_ and *a*_1_ in the 7EMM. A strong correlation was observed between relaxation force values as well as between relaxation times. In contrast, the relationships between the parameters F_1_–F_c_ and *a*_1_–*a*_3_ were noticeably weaker, with correlation coefficients ranging from 0.51 to 0.72. However, a closer analysis, despite the presence of strong correlations, revealed an additional insight (Figure 12).

The above data confirm the observed significant change in the relationship between relaxed force values within the moisture range of 12–14%. For hard wheat cultivars at moisture levels up to 12%, increases in force during the longest relaxation period were accompanied by relatively small increases in force values during shorter phases of the relaxation process. In contrast, when moisture content exceeded 14%, the opposite trend was observed: minor changes in the F_1_ force were accompanied by substantial increases in forces associated with shorter relaxation times (F_1_ and F_2_). This pattern was observed in both presented models.

In the case of soft cultivars, the relationship between the forces corresponding to long and shorter relaxation times was more linear, although faster increases in relaxed force values during shorter time intervals became evident at moisture levels above 12%.

It should be noted that the presented averages and trends are based on approximately 22,000 models generated for each of the three analyzed model types. This extensive dataset encompasses diverse grain types and moisture levels, as well as various applied loads and relaxation times, with 20 independent replications performed (using 20 individual kernels).

While the current findings provide a robust basis for statistically justified conclusions, expanding future studies to a broader spectrum of wheat cultivars with well-defined characteristics would be beneficial. Future work will focus on correlating these results with standardized hardness assessment methods, such as the Single-Kernel Characterization System [32], Particle Size Index [39] or others, to improve varietal classification and better understand grain behavior during processing.

Furthermore, explicitly incorporating load magnitude and relaxation time as independent variables in subsequent multi-factor analyses will allow us to validate the observed regularities. This approach will also help to identify specific testing conditions under which these viscoelastic effects are either amplified or diminished.

## 5. Conclusions

A novel aspect of our work is the comparative application of three Maxwell model configurations (3-, 5-, and 7-element) to the same dataset. This approach allowed us to observe that increasing model complexity tends to reduce differences in parameter values related to wheat type and moisture content, suggesting a trade-off between model accuracy and interpretability. This insight can guide researchers in selecting appropriate viscoelastic models for grain rheology.

Increasing moisture content consistently led to higher magnitudes of force decay (spring constants) and faster relaxation rates (dashpot constants), reflecting softer and more plastic kernel properties. Distinct relaxation behaviors were observed between hard and soft wheat varieties. Hard wheat varieties showed higher residual forces and slower relaxation rates, confirming their greater structural resistance compared to soft types.

The variability in model parameters for soft and hard wheat cultivars depending on moisture content indicated the critical importance of this factor in comparing grain responses during the load relaxation process. It also revealed significant changes in this response within the 12–14% moisture range, which were particularly pronounced for hard cultivars.

Overall, our findings provide a comprehensive understanding of how moisture and wheat variety influence kernel viscoelastic behavior and emphasize the need for further research on the effects of loading conditions and relaxation times. Such investigations will enhance modeling accuracy and contribute to better predictions of grain mechanical properties.

## Figures and Tables

**Table 1:** Chemical composition of wheat used in the study [28].

Wheat	Parameter	Unit	Result
HARD1	Protein content (d.b.)	%	15.2 ± 0.6
	Starch content (d.b.)	%	63.7 ± 1.9
	Wet gluten content	%	35.2 ± 3.6
	Zeleny index	mL	61.8 ± 10.7
HARD2	Protein content (d.b.)	%	15.0 ± 0.7
	Starch content (d.b.)	%	64.6 ± 2.0
	Wet gluten content	%	33.7 ± 3.4
	Zeleny index	mL	64.6 ± 11.7
SOFT1	Protein content (d.b.)	%	11.8 ± 0.5
	Starch content (d.b.)	%	70.3 ± 2.2
	Wet gluten content	%	26.2 ± 2.6
	Zeleny index	mL	34.0 ± 5.9
SOFT2	Protein content (d.b.)	%	11.9 ± 0.5
	Starch content (d.b.)	%	71.1 ± 2.2
	Wet gluten content	%	25.4 ± 2.6
	Zeleny index	mL	34.5 ± 6.0

**Table 2:** Root Mean Square Error (RMSE) and R^2^ for the applied Maxwell models in relation to the moisture content of wheat.

Parameter	Model	Moisture Content [%]						
		8	10	12	14	16	18	20
RMSE	3EMM	3.92	3.9	3.56	2.4	2.33	1.77	1.64
	5EMM	3.90	3.88	3.53	2.32	2.24	1.61	1.47
	7EMM	3.87	3.85	3.51	2.31	2.23	1.58	1.44
R^2^	3EMM	0.9252	0.9305	0.9293	0.9138	0.8999	0.8920	0.8920
	5EMM	0.9914	0.9928	0.9914	0.9890	0.9872	0.9872	0.9872
	7EMM	0.9975	0.9986	0.9968	0.9972	0.9970	0.9977	0.9977

3EMM—three-element Maxwell model, 5EMM—five-element Maxwell model, 7EMM—seven-element Maxwell model.

**Table 3:** Correlation matrix between the parameters of the 5EMM.

Variable	Correlations in 5EMM (Marked Correlations Are Significant at *p* < 0.05, N = 14; Averages for Wheat Type and Moisture)				
	F_1_	F_2_	F_c_	*a*_1_	*a*_2_
F_1_	-				
				
F_2_	0.9633	-			
	*p* = 0.000				
F_c_	−0.9704	−0.9933	-		
	*p* = 0.000	*p* = 0.000			
*a*_1_	0.8668	0.9643	−0.9516	-	
	*p* = 0.000	*p* = 0.000	*p* = 0.000		
*a*_2_	0.7351	0.8619	−0.8702	0.9399	-
	*p* = 0.003	*p* = 0.000	*p* = 0.000	*p* = 0.000	

**Table 4:** Correlation matrix between the parameters of the 7EMM.

Variable	Correlations in 7EMM (Marked Correlations Are Significant at *p* < 0.05; N = 14; Averages for Wheat Type and Moisture)						
	F_1_	F_2_	F_3_	F_c_	*a*_1_	*a*_2_	*a*_3_
F_1_	-						
						
F_2_	0.9602	-					
	*p* = 0.000						
F_3_	0.9374	0.9915	-				
	*p* = 0.000	*p* = 0.000					
F_c_	−0.9522	−0.9906	−0.9974	-			
	*p* = 0.000	*p* = 0.000	*p* = 0.000				
*a*_1_	0.5083	0.6140	0.6081	−0.6036	-		
	*p* = 0.063	*p* = 0.019	*p* = 0.021	*p* = 0.022			
*a*_2_	0.5653	0.6364	0.6527	−0.6623	0.9450	-	
	*p* = 0.035	*p* = 0.014	*p* = 0.011	*p* = 0.010	*p* = 0.000		
*a*_3_	0.6079	0.6874	0.7150	−0.7256	0.8559	0.9483	-
	*p* = 0.021	*p* = 0.007	*p* = 0.004	*p* = 0.003	*p* = 0.000	*p* = 0.000	

## Data Availability

The original contributions presented in this study are included in the article/Supplementary Material. Further inquiries can be directed to the corresponding authors.

## Supplementary Materials

The following supporting information can be downloaded at: https://www.mdpi.com/article/10.3390/ma19122592/s1, Table S1: Basics statistics for the parameters of the 3EMM model; Table S2: Basics statistics for the parameters of the 5EMM model; Table S3: Basics statistics for the parameters of the 7EMM model; Table S4. Analysis of variance for the effect of wheat moisture and hardness on the parameters of the 3EMM model; Table S5. Analysis of variance for the effect of wheat moisture and hardness on the parameters of the 5EMM model; Table S6. Analysis of variance for the effect of wheat moisture and hardness on the parameters of the 7EMM model.

## References

[1] Gunasekaran S., Ak M.M. (2000). Dynamic oscillatory shear testing of foods—Selected applications. Trends Food Sci. Technol..

[2] Sozer N., Kaya A., Dalgic A.C. (2008). The Effect of Resistant Starch Addition on Viscoelastic Properties of Cooked Spaghetti. J. Texture Stud..

[3] Sozer N., Dalgic A.C. (2007). Modelling of Rheological Characteristics of Various Spaghetti Types. Eur. Food Res. Technol..

[4] Filipčev B.V. (2014). Texture and Stress Relaxation of Spelt-Amaranth Composite Breads. Food Feed Res..

[5] Steffe F.S. Rheological Methods in Food Process Engineering—James Freeman Steffe—Google Books. https://books.google.pl/books?hl=en&lr=&id=LrrdONuST9kC&oi=fnd&pg=PR3&dq=Rheological+methods+in+food+process+engineering.&ots=k0SRjyb9g5&sig=RpgV4H_4fuDllzsfYlPhl6SLn6U&redir_esc=y#v=onepage&q=Rheological%20methods%20in%20food%20process%20engineering.&f=false.

[6] Del Nobile M.A., Chillo S., Falcone P.M., Laverse J., Pati S., Baiano A. (2007). Textural Changes of Canestrello Pugliese Cheese Measured during Storage. J. Food Eng..

[7] Hernández Z.J.E., Figueroa J.D.C., Rayas-Duarte P., Martínez-Flores H.E., Arámbula G.V., Luna G.B., Peña R.J. (2012). Influence of High and Low Molecular Weight Glutenins on Stress Relaxation of Wheat Kernels and the Relation to Sedimentation and Rheological Properties. J. Cereal Sci..

[8] Figueroa J.D.C., Hernández Z.J.E., Rayas-Duarte P., Peña R.J. (2013). Stress Relaxation and Creep Recovery Tests Performed on Wheat Kernels versus Doughs: Influence of Glutenins on Rheological and Quality Properties. Cereal Foods World.

[9] Figueroa J.D.C., Manuel C.I., Hernández-Estrada Z.J., Ramírez-Wong B. (2012). Stress Relaxation of Wheat Kernels and Their Relationship with Milling, Rheological, and Breadmaking Quality of Wheat. Cereal Chem..

[10] Bargale P.C., Irudayaraj J., Marquis B. (1995). Studies on Rheological Behaviour of Canola and Wheat. J. Agric. Eng. Res..

[11] Wang P., Wang L.J., Li D., Huang Z.G., Adhikari B., Chen X.D. (2017). The Stress-Relaxation Behavior of Rice as a Function of Time, Moisture and Temperature. Int. J. Food Eng..

[12] Waananen K.M., Okos M.R. (1992). Stress-Relaxation Properties of Yellow-Dent Corn Kernels Under Uniaxial Loading. Trans. ASAE.

[13] Sheng S.Y., Wang L.J., Li D., Mao Z.H., Adhikari B. (2014). Viscoelastic Behavior of Maize Kernel Studied by Dynamic Mechanical Analyzer. Carbohydr. Polym..

[14] Pappas G., Skinner G.E., Rao V.N.M. (1988). Effect of Imposed Strain and Moisture Content on Some Viscoelastic Characteristics of Cowpeas (Vigna Unguiculata). J. Agric. Eng. Res..

[15] Liu M., Haghighi K., Stroshine R.L., Ting E.C. (1990). Mechanical Properties of the Soybean Cotyledon and Failure Strength of Soybean Kernels. Trans. ASAE.

[16] Bargale P.C., Irudayaraj J.M., Marquis B. (1994). Some Mechanical Properties and Stress Relaxation Characteristics of Lentils. Can. Agric. Eng..

[17] Wu M.Y., Chang Y.H., Shiau S.Y., Chen C.C. (2020). Rheology of Fiber-Enriched Steamed Bread: Stress Relaxation and Texture Profile Analysis. J. Food Drug Anal..

[18] Yildiz Ö., Yurt B., Baştürk A., Toker Ö.S., Yilmaz M.T., Karaman S., Daǧlioǧlu O. (2013). Pasting Properties, Texture Profile and Stress-Relaxation Behavior of Wheat Starch/Dietary Fiber Systems. Food Res. Int..

[19] Ben Z., Sun X., Bai Y., Yang D., Dong Y., Chen K. (2025). Research on the Densification Process and Constitutive Model of Gluten. J. Food Eng..

[20] Ozturk O.K., Takhar P.S. (2017). Stress Relaxation Behavior of Oat Flakes. J. Cereal Sci..

[21] Shellhammer T.H., Rumsey T.R., Krochta J.M. (1997). Viscoelastic Properties of Edible Lipids. J. Food Eng..

[22] Mancini M., Moresi M., Rancini R. (1999). Mechanical Properties of Alginate Gels: Empirical Characterisation. J. Food Eng..

[23] Fincan M., Dejmek P. (2003). Effect of Osmotic Pretreatment and Pulsed Electric Field on the Viscoelastic Properties of Potato Tissue. J. Food Eng..

[24] Lachowicz K., Sobczak M., Gajowiecki L., Zych A. (2003). Effects of Massaging Time on Texture, Rheological Properties, and Structure of Three Pork Ham Muscles. Meat Sci..

[25] Limanond B., Castell-Perez M.E., Moreira R.G. (2002). Modeling the Kinetics of Corn Tortilla Staling Using Stress Relaxation Data. J. Food Eng..

[26] Lewicki P.P. (2004). Water as the Determinant of Food Engineering Properties. A Review. J. Food Eng..

[27] Łysiak G. (2007). Influence of Moisture on Rheological Characteristics of the Kernel of Wheat. Acta Agrophysica.

[28] Kadhim J., Aridhee A., Łysiak G., Kulig R., Wójcik M., Panasiewicz M. (2019). The Effect of Wheat Moisture and Hardness on the Parameters of the Peleg and Normand Model during Relaxation of Single Kernels at Variable Initial Loading. Sustainability.

[29] Łysiak G., Al Aridhee J.K., Kulig R., Różyło R., Wójcik M. (2021). Examination of the Peleg and Normand Equation during Relaxation of Wheat: The Effect of Holding Time. J. Texture Stud..

[30] Bhattacharya S. (2010). Stress Relaxation Behaviour of Moth Bean Flour Dough: Product Characteristics and Suitability of Model. J. Food Eng..

[31] Khazaei J., Mann D.D. (2005). Effects of Moisture Content and Number of Loadings on Force Relaxation Behaviour of Chickpea Kernels. Int. Agrophys..

[32] Osborne B.G., Anderssen R.S. (2003). Single-Kernel Characterization Principles and Applications. Cereal Chem..

[33] Markowski M., Ratajski A., Konopko H., Zapotoczny P., Majewska K. (2006). Rheological Behavior of Hot-Air-Puffed Amaranth Seeds. Int. J. Food Prop..

[34] Sarig Y., Orlovsky S. (1974). Viscoelastic Properties of Shamouti Oranges. J. Texture Stud..

[35] Herak D., Kabutey A., Choteborsky R., Petru M., Sigalingging R. (2015). Mathematical Models Describing the Relaxation Behaviour of *Jatropha curcas* L. Bulk Seeds under Axial Compression. Biosyst. Eng..

[36] Liu M., Haghighi K., Stroshine R.L. (1989). Viscoelastic Characterization of the Soybean Seedcoat. Trans. Am. Soc. Agric. Eng..

[37] Zheng Z., Ren L., Fu H., Yang P., Lv L., Xu J., Yang D. (2023). Investigation on Relaxation Properties of Maize Kernels Based on the Multicomponent Structure. Powder Technol..

[38] Ponce-García N., Ramírez-Wong B., Torres-Chávez P.I., Figueroa-Cárdenas J.d.D., Serna-Saldívar S.O., Cortez-Rocha M.O., Escalante-Aburto A. (2017). Evaluation of Visco-Elastic Properties of Conditioned Wheat Kernels and Their Doughs Using a Compression Test under Small Strain. J. Sci. Food Agric..

[39] Dziki D., Krajewska A., Findura P. (2024). Particle Size as an Indicator of Wheat Flour Quality: A Review. Processes.

