# Supplementary material for: Application of the Generalized Maxwell Model for Single-Kernel Relaxation Experiments—Part 1: Effect of Wheat Type and Moisture Content
Source: Materials (Basel). 2026 Jun 16;19(12):2592. doi: 10.3390/ma19122592 (PMC13304345; doi:10.3390/ma19122592)
Supplement: Supplementary file 1 [file materials-19-02592-s001.zip › materials-4346412-supplementary.pdf]

The statistics presented below are based on analyses performed for the following independent variables: wheat variety (hardness), moisture content, initial load, and relaxation time. The tables include data only for variety and moisture content.

Table S1. Basics statistics for the parameters of the 3EMM model

Effect	Level of factor	Wheat variety				Moisture [%]						
		HARD1	HARD2	SOFT1	SOFT2	8	10	12	14	16	18	20
F_1 [N]	Mean	10.24	10.29	9.93	10.06	7	7.48	7.74	10.24	11.97	12.37	13.99
	Std.Dev.	4.18	4.52	4.07	4.15	1.94	2.09	2.06	3.5	4.11	4.24	4.49
	Std.Err.	0.0562	0.0608	0.0546	0.0558	0.0347	0.0373	0.0367	0.0620	0.0729	0.0750	0.0795
	**CV (%)**	**40.82**	**43.93**	**40.99**	**41.25**	**27.71**	**27.94**	**26.61**	**34.18**	**34.34**	**34.28**	**32.09**
F_c [N]	Mean	20.91	20.78	20.45	19.45	29.27	27.27	25.94	19.39	15.1	12.49	13.65
	Std.Dev.	10.3	10.23	8.97	8.57	8.66	8.58	8.36	6.41	5.8	5.1	5.39
	Std.Err.	0.1383	0.1374	0.1203	0.1151	0.1548	0.1530	0.1488	0.1135	0.1027	0.0904	0.0954
	**CV (%)**	**49.26**	**49.23**	**43.86**	**44.06**	**29.59**	**31.46**	**32.23**	**33.06**	**38.41**	**40.83**	**39.49**
a_1 [s^{-1}]	Mean	0.164	0.16	0.176	0.177	0.141	0.141	0.143	0.167	0.189	0.199	0.204
	Std.Dev.	0.162	0.158	0.177	0.181	0.134	0.147	0.147	0.171	0.184	0.189	0.192
	Std.Err.	0.0022	0.0021	0.0024	0.0024	0.0024	0.0026	0.0026	0.0030	0.0033	0.0033	0.0034
	**CV (%)**	**98.78**	**98.75**	**100.57**	**102.26**	**95.04**	**104.26**	**102.80**	**102.40**	**97.35**	**94.97**	**94.12**

Table S2. Basics statistics for the parameters of the 5EMM model

Effect	Level of factor	Wheat variety				Moisture [%]						
		HARD1	HARD2	SOFT1	SOFT2	8	10	12	14	16	18	20
F_1 [N]	Mean	7.15	7.18	6.72	6.86	5.15	5.56	5.8	7.42	7.98	8.46	8.55
	Std.Dev.	2.5	2.79	2.35	2.39	1.36	1.38	1.35	2.33	2.49	2.57	2.87
	Std.Err.	0.0341	0.0380	0.0320	0.0326	0.0245	0.0249	0.0243	0.0416	0.0447	0.0466	0.0524
	**CV (%)**	**34.97**	**38.86**	**34.97**	**34.84**	**26.41**	**24.82**	**23.28**	**31.40**	**31.20**	**30.38**	**33.57**
F_2 [N]	Mean	9.42	9.49	9.56	9.89	5.71	6.32	6.59	9.59	12.05	13.36	13.67
	Std.Dev.	4.77	4.96	4.56	4.6	1.98	2.39	2.25	3.81	4.23	4.46	4.32
	Std.Err.	0.0651	0.0676	0.0620	0.0627	0.0357	0.0431	0.0406	0.0681	0.0759	0.0809	0.0787
	**CV (%)**	**50.64**	**52.27**	**47.70**	**46.51**	**34.68**	**37.82**	**34.14**	**39.73**	**35.10**	**33.38**	**31.60**
F_c [N]	Mean	20.33	20.21	19.75	18.99	28.69	26.65	25.33	18.69	14.4	12.63	12.04
	Std.Dev.	10.14	10.08	9.04	8.46	8.49	8.42	8.23	6.23	5.52	4.97	5.02
	Std.Err.	0.1382	0.1372	0.1228	0.1153	0.1525	0.1512	0.1481	0.1112	0.0990	0.0902	0.0916
	**CV (%)**	**49.88**	**49.88**	**45.77**	**44.55**	**29.59**	**31.59**	**32.49**	**33.33**	**38.33**	**39.35**	**41.69**
a_1 [s^{-1}]	Mean	0.078	0.076	0.08	0.081	0.071	0.07	0.071	0.077	0.084	0.087	0.089
	Std.Dev.	0.071	0.068	0.074	0.074	0.062	0.062	0.066	0.073	0.078	0.078	0.078
	Std.Err.	0.0010	0.0009	0.0010	0.0010	0.0011	0.0011	0.0012	0.0013	0.0014	0.0014	0.0014
	**CV (%)**	**91.03**	**89.47**	**92.50**	**91.36**	**87.32**	**88.57**	**92.96**	**94.81**	**92.86**	**89.66**	**87.64**
a_2 [s^{-1}]	Mean	1.058	1.061	1.208	1.243	0.912	0.953	1.017	1.145	1.338	1.328	1.313
	Std.Dev.	0.896	0.798	0.973	0.99	0.622	0.641	0.758	1.029	1.145	1.07	0.922
	Std.Err.	0.0122	0.0109	0.0132	0.0135	0.0112	0.0115	0.0136	0.0184	0.0205	0.0194	0.0168
	**CV (%)**	**84.69**	**75.21**	**80.55**	**79.65**	**68.20**	**67.26**	**74.53**	**89.87**	**85.58**	**80.57**	**70.22**

Table S3. Basics statistics for the parameters of the 7EMM model

Effect	Level of factor	Wheat variety				Moisture [%]						
		HARD1	HARD2	SOFT1	SOFT2	8	10	12	14	16	18	20
F_1 [N]	Mean	6.03	6.09	5.69	5.78	4.48	4.92	5.19	6.37	6.63	6.92	6.95
	Std.Dev.	1.94	2.26	1.84	1.86	1.16	1.18	1.15	1.78	2.01	2.14	2.39
	Std.Err.	0.0275	0.0323	0.0261	0.0265	0.0216	0.0221	0.0213	0.0336	0.0381	0.0410	0.0459
	**CV (%)**	**32.17**	**37.11**	**32.34**	**32.18**	**25.89**	**23.98**	**22.16**	**27.94**	**30.32**	**30.92**	**34.39**
F_2 [N]	Mean	5.63	5.61	5.47	5.56	3.7	3.91	4.18	5.72	6.77	7.4	7.6
	Std.Dev.	2.37	2.53	2.31	2.19	1.03	1.08	1.23	1.83	2.11	2.27	2.35
	Std.Err.	0.0336	0.0361	0.0328	0.0314	0.0190	0.0203	0.0227	0.0345	0.0400	0.0434	0.0451
	**CV (%)**	**42.10**	**45.10**	**42.23**	**39.39**	**27.84**	**27.62**	**29.43**	**31.99**	**31.17**	**30.68**	**30.92**
F_3 [N]	Mean	7.4	7.48	7.66	7.99	4.23	4.95	5.09	7.86	9.84	10.83	11.14
	Std.Dev.	4.21	4.39	4.01	4.07	1.89	2.32	2.15	3.54	3.88	3.93	3.75
	Std.Err.	0.0597	0.0625	0.0567	0.0581	0.0350	0.0433	0.0397	0.0666	0.0736	0.0751	0.0719
	**CV (%)**	**56.89**	**58.69**	**52.35**	**50.94**	**44.68**	**46.87**	**42.24**	**45.04**	**39.43**	**36.29**	**33.66**
F_c [N]	Mean	19.84	19.48	19.27	18.43	28	25.94	24.82	18.16	13.63	11.76	11.23
	Std.Dev.	10.09	10.04	8.93	8.36	8.26	8.19	8.11	6	5.27	4.68	4.77
	Std.Err.	0.1428	0.1430	0.1264	0.1193	0.1525	0.1530	0.1494	0.1128	0.0999	0.0895	0.0914
	**CV (%)**	**50.86**	**51.54**	**46.34**	**45.36**	**29.50**	**31.57**	**32.68**	**33.04**	**38.66**	**39.80**	**42.48**
a_1 [s^{-1}]	Mean	0.042	0.041	0.044	0.043	0.042	0.039	0.042	0.043	0.044	0.044	0.043
	Std.Dev.	0.038	0.037	0.039	0.039	0.037	0.034	0.039	0.039	0.04	0.04	0.039
	Std.Err.	0.0005	0.0005	0.0006	0.0005	0.0007	0.0006	0.0007	0.0007	0.0008	0.0008	0.0008
	**CV (%)**	**90.48**	**90.24**	**88.64**	**90.70**	**88.10**	**87.18**	**92.86**	**90.70**	**90.91**	**90.91**	**90.70**
a_2 [s^{-1}]	Mean	0.34	0.33	0.357	0.353	0.325	0.306	0.345	0.361	0.365	0.361	0.356
	Std.Dev.	0.271	0.269	0.278	0.277	0.26	0.237	0.289	0.283	0.285	0.281	0.275
	Std.Err.	0.0038	0.0038	0.0039	0.0040	0.0048	0.0044	0.0053	0.0053	0.0054	0.0054	0.005273
	**CV (%)**	**79.71**	**81.52**	**77.87**	**78.47**	**80.00**	**77.45**	**83.77**	**78.39**	**78.08**	**77.84**	**77.25**
a_3 [s^{-1}]	Mean	2.97	2.85	3.25	3.34	2.49	2.3	3.08	3.29	3.83	3.48	3.33
	Std.Dev.	3.76	5.06	3.95	4.27	3.84	4.07	5.18	2.93	5.24	3.67	4.38
	Std.Err.	0.0532	0.0721	0.0559	0.0610	0.0710	0.0760	0.0955	0.0551	0.0994	0.0703	0.0840
	CV (%)	**126.60**	**177.54**	**121.54**	**127.84**	**154.22**	**176.96**	**168.18**	**89.06**	**136.81**	**105.46**	**131.53**

Table S4. Analysis of variance for the effect of wheat moisture and hardness on the parameters of the 3EMM model.

Three element Maxwell model 3EMM					
	F_1				
Effect	SS	DF	MS	F	P
Intercept	2273102	1	2273102	203920.1	0.000
Hardness (H)	363	1	363	32.6	0.000
Moisture content MC	145406	6	24234	2174.1	0.000
H × MC	6300	6	1050	94.2	0.000
Error	247453	22199	11		
	F_c				
Effect	SS	DF	MS	F	P
Intercept	9290254	1	9290254	191481.4	0.000
Hardness (H)	4792	1	4792	98.8	0.000
Moisture content MC	930324	6	155054	3195.8	0.000
H × MC	21183	6	3531	72.8	0.000
Error	1077046	22199	49		
	a_1				
Effect	SS	DF	MS	F	p
Intercept	636.5643	1	636.5643	22658.87	0.000
Hardness (H)	1.1931	1	1.1931	42.47	0.000
Moisture content MC	15.1824	6	2.5304	90.07	0.000
H × MC	0.1191	6	0.0199	0.71	0.644
Error	623.6450	22199	0.0281		

H – hardness, MC – moisture content, SS – sum of squares, DF – degree of freedom, MS – mean square, F– statistics, P – value

Table S5. Analysis of variance for the effect of wheat moisture and hardness on the parameters of the 5EMM model.

Five element Maxwell model 5EMM					
	F_1				
Effect	SS	DF	MS	F	p
Intercept	1056233	1	1056233	237571.5	0.000
Hardness (H)	731	1	731	164.4	0.000
Moisture content MC	38850	6	6475	1456.4	0.000
H × MC	2093	6	349	78.5	0.000
Error	96019	21597	4		
	F_2				
Effect	SS	DF	MS	F	p
Intercept	1998557	1	1998557	164820.4	0.000
Hardness (H)	401	1	401	33.0	0.000
Moisture content MC	220003	6	36667	3023.9	0.000
H × MC	2114	6	352	29.1	0.000
Error	261878	21597	12		
	F_c				
Effect	SS	DF	MS	F	p
Intercept	8457221	1	8457221	182090.8	0.000
Hardness (H)	4436	1	4436	95.5	0.000
Moisture content MC	918990	6	153165	3297.8	0.000
H × MC	14743	6	2457	52.9	0.000
Error	1003074	21597	46		
	a_1				
Effect	SS	DF	MS	F	p
Intercept	133.0625	1	133.0625	26202.68	0.000
Hardness (H)	0.0625	1	0.0625	12.30	0.000
Moisture content MC	1.1887	6	0.1981	39.01	0.000
H × MC	0.0170	6	0.0028	0.56	0.764
Error	109.6739	21597	0.0051		
	a_2				
Effect	SS	DF	MS	F	p
Intercept	28270.62	1	28270.62	34888.52	0.000
Hardness (H)	148.84	1	148.84	183.69	0.000
Moisture content MC	636.24	6	106.04	130.86	0.000
H × MC	49.86	6	8.31	10.26	0.000
Error	17500.33	21597	0.81		

H – hardness, MC – moisture content, SS – sum of squares, DF – degree of freedom, MS – mean square, F– statistics, P – value

Table S6. Analysis of variance for the effect of wheat moisture and hardness on the parameters of the 7EMM model.

Seven element Maxwell model 7EMM					
	F_1				
Effect	SS	DF	MS	F	p
Intercept	695873.9	1	695873.9	234404.7	0.000
Hardness (H)	517.1	1	517.1	174.2	0.000
Moisture content MC	18153.6	6	3025.6	1019.2	0.000
H × MC	1153.1	6	192.2	64.7	0.000
Error	58836.4	19819	3.0		
	F_2				
Effect	SS	DF	MS	F	p
Intercept	624614.5	1	624614.5	201373.7	0.000
Hardness (H)	53.5	1	53.5	17.2	0.000
Moisture content MC	48365.2	6	8060.9	2598.8	0.000
H × MC	665.6	6	110.9	35.8	0.000
Error	61473.9	19819	3.1		
	F_3				
Effect	SS	DF	MS	F	p
Intercept	1178056	1	1178056	119323.6	0.000
Hardness (H)	740	1	740	74.9	0.000
Moisture content MC	148869	6	24812	2513.1	0.000
H × MC	1430	6	238	24.1	0.000
Error	195669	19819	10		
	F_c				
Effect	SS	DF	MS	F	p
Intercept	7217596	1	7217596	164442.7	0.000
Hardness (H)	3288	1	3288	74.9	0.000
Moisture content MC	865156	6	144193	3285.2	0.000
H × MC	14846	6	2474	56.4	0.000
Error	869881	19819	44		
	a_1				
Effect	SS	DF	MS	F	p
Intercept	35.77212	1	35.77212	24377.98	0.000
Hardness (H)	0.00816	1	0.00816	5.56	0.018
Moisture content MC	0.06111	6	0.01018	6.94	0.000
H × MC	0.01833	6	0.00305	2.08	0.052
Error	29.08230	19819	0.00147		
	a_2				
Effect	SS	DF	MS	F	p
Intercept	2366.860	1	2366.860	31776.22	0.000
Hardness (H)	2.056	1	2.056	27.60	0.000
Moisture content MC	8.556	6	1.426	19.14	0.000
H × MC	1.894	6	0.316	4.24	0.000
Error	1476.224	19819	0.074		
	a_3				
Effect	SS	DF	MS	F	p
Intercept	192774.1	1	192774.1	10641.88	0.000
Hardness (H)	752.2	1	752.2	41.53	0.000
Moisture content MC	5056.6	6	842.8	46.52	0.000
H × MC	828.5	6	138.1	7.62	0.000
Error	359014.7	19819	18.1		

H – hardness, MC – moisture content, SS – sum of squares, DF – degree of freedom, MS – mean square, F– statistics, P – value
